# Declines the Honor

**Published:** 1885-11

**Authors:** John W. Elston, Emory Lanphear

**Affiliations:** Editor of Index; Kansas City, Mo.; Editor of Index; Kansas City, Mo.


### CORRESPONDENCE.

### DECLINES THE HONOR.

IT will be remembered that the *New York Medical Record* asserted that "the profession of America almost unanimously condemn the action of the American Medical Association," and cited *eighteen* medical journals—out of the nearly two hundred —as so opposing, in support of the assertion. Amongst these eighteen was the *Kansas City Medical Index.* The following from the editors of that staunch and progressive periodical will show how much authority the *Record* had for using the name of the *Index:*

KANSAS CITY, MO., October 17, 1885.

*Dear Dr. Daniel:*

Please correct statement in last issue of your highly esteemed journal, that the *Kansas City Medical Index* is *opposed* to the action of the American Medical Association. We do *not* belong to the *New York Record-Columbus Journal* clique, as you will see by our October number, mailed you this week. (See editorial "A double Correction.) We sincerely wish the International Congress to be a success, and join you in wanting *all* sections of the country represented therein.

With best wishes, we are

Yours fraternally,

JOHN W. ELSTON,

EMORY LANPHEAR,

Editors of *Index.*

The name of the *Medical Journal and Examiner* was used also without authority in the same list,—the editor being one of the chairmen of sections under the new appointments.

Again: We had classified *Leonard's Illustrated* as neutral—being of those who took no part in the controversy. Dr. Leonard requests us to make the correction, and to put him and his *Illustrated* [and illustrious] where it ought to be, on the side of "right." We do so with much pleasure. This brings the *Record* down to fifteen journals out of the whole, and puts our list up to sixteen of the very foremost and most independent and able and *honest* journals in America as endorsing *unequivocally* the action of the American Medical Association with reference to the International Congress. If the *Record* and the *Columbus Journal* are capable of blushing, *now's* their time, or *never*.

